# Aberrant rhythmic expression of *cryptochrome2* regulates the radiosensitivity of rat gliomas

**DOI:** 10.18632/oncotarget.20835

**Published:** 2017-09-12

**Authors:** Wang Fan, Li Caiyan, Zhu Ling, Zhao Jiayun

**Affiliations:** ^1^ Department of Neurosurgery, The First People's Hospital of Jingmen, Jingmen 448000, China; ^2^ The Center of Cancer Prevention, The Second People's Hospital of Jingmen, Jingmen 448000, China

**Keywords:** circadian rhythm, *cry2*, glioma, radiotherapeutic sensitivity

## Abstract

In this study, we investigated the role of the clock regulatory protein cryptochrome 2 (Cry2) in determining the radiosensitivity of C6 glioma cells in a rat model. We observed that Cry2 mRNA and protein levels showed aberrant rhythmic periodicity of 8 h in glioma tissues, compared to 24 h in normal brain tissue. Cry2 mRNA and protein levels did not respond to irradiation in normal tissues, but both were increased at the ZT4 (low Cry2) and ZT8 (high Cry2) time points in gliomas. Immunohistochemical staining of PCNA and TUNEL assays demonstrated that high Cry2 expression in glioma tissues was associated with increased cell proliferation and decreased apoptosis. Western blot analysis showed that glioma cell fate was independent of p53, but was probably dependent on p73, which was more highly expressed at ZT4 (low Cry2) than at ZT8 (high Cry2). Levels of both p53 and p73 were unaffected by irradiation in normal brain tissues. These findings suggest aberrant rhythmic expression of Cry2 influence on radiosensitivity in rat gliomas.

## INTRODUCTION

The daily light-dark cycle synchronizes the master circadian pacemaker, which is located in the suprachiasmatic nuclei (SCN) of the brain that synchronizes the organism's central clock as well as the peripheral clocks in each cell [1]. The molecular mechanisms of circadian oscillation in the SCN and peripheral cells are based on a negative transcriptional-translational feedback loop regulated by the core clock genes [2]. *CLOCK* and *BMAL1* proteins associate as heterodimers and bind to the E-box enhancer elements, thereby inducing the expression of *Period* (*Per1, Per2, Per3*) and *Cryptochrome* (*Cry1, Cry2*) genes [3]. The Period and Cryptochrome proteins associate into multimeric complexes and repress transactivation by *CLOCK/BMAL1* by a negative feedback loop, thereby negatively regulating *Cry1* and *Cry2* expression [3–4].

The circadian system regulates cellular growth, proliferation and apoptosis [5–6]. In fact, circadian rhythms regulate diverse physiological processes such as hormone secretion, metabolism, cell proliferation and apoptosis [7–8]. Therefore, deregulation of the circadian clock alters the expression of clock-controlled genes and can influence cell and organ function.

Cryptochrome 2 (CRY2) is one of the circadian clock proteins, which is involved in DNA-damage check-point control and cell-cycle progression [9, 10]. CRY mutations increase sensitivity to apoptosis by genotoxic agents and protect p53-mutant mice from the early onset of cancer [11]. Also, breast cancer cells with reduced CRY2 demonstrate increased mutagen-induced DNA damage [12–14]. These studies suggest that CRY2 regulated DNA damage and cell apoptosis [15]. Also, CRY2 was overexpressed in chemoresistant colorectal cancer patient samples suggesting that CRY2 was a potential therapeutic target in cancer treatment [15]. In this study, we investigated if Cry2 regulated glioma proliferation and apoptosis treatmented by irradiation.

## RESULTS

### Aberrant *Cry 2* mRNA and protein expression and rhythmicity in glioma brain tissues

**Figure 1:**
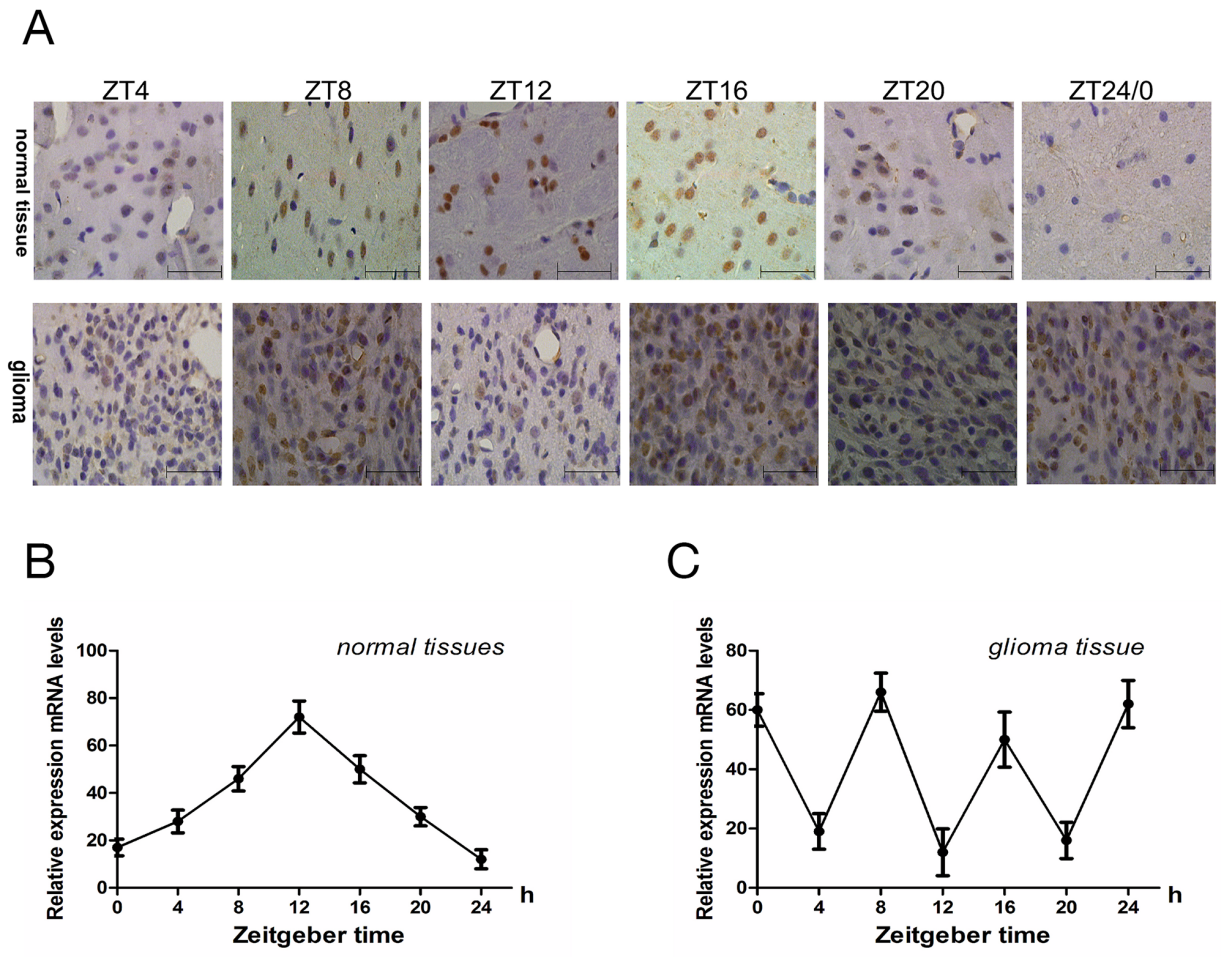
The rhythmic expression of Cry 2 mRNA and protein in the rat glioma model **(A)** Immunohistochemical analysis of Cry2 protein expression in glioma and normal brain samples at ZT0, ZT4, ZT8, ZT12, ZT16 and ZT20 time points. **(B, C)** QRT-PCR analysis of cry2 mRNA levels in normal and glioma brain at ZT0, ZT4, ZT8, ZT12, ZT16 and ZT20 time points. Cry2 mRNA and protein expression changed according to a 24h-cycle (mRNA [F=8.34, P<0.001]; protein [F=9.75, P<0.001]) in normal brain tissues and a 8h-cycle in (mRNA [F=15.04, P<0.001]; protein [F=12.09, P<0.001]) in glioma brain tissues. Data are the mean and standard deviation of three independent experiments.

C*ry2*mRNA expression in normal and glioma brain tissues samples from rats was analyzed at ZT0 (ZT24), ZT4, ZT8, ZT12, ZT16 and ZT20 time points by qRT-PCR. First, we observed that *cry2* mRNA expression in normal brains was low at ZT0 and gradually increased at ZT4 and ZT8 and peaked at ZT12 before subsequently decreasing gradually at ZT16, ZT20 and ZT24 (Figure 1B). However, in the glioma tissues, *cry2* mRNA expression was aberrant with two 2 peaks at ZT8 and ZT16 and low at ZT4, ZT12 and ZT20 (Figure 1C). Immunohistochemical staining of Cry2 protein in the glioma and normal brain tissues showed similar pattern as cry2 mRNA (Figure 1A). Statistical analysis showed that Cry2 mRNA (F=8.34, p<0.001) and protein (F=9.75, p<0.001) expression in normal brain tissues followed a 24h periodic cycle, whereas the Cry2 mRNA (F=15.04, p<0.001) and protein (F=12.09, p<0.001) followed a 8h periodic cycle.

### Irradiation affects *cry 2* gene expression in gliomas

**Figure 2:**
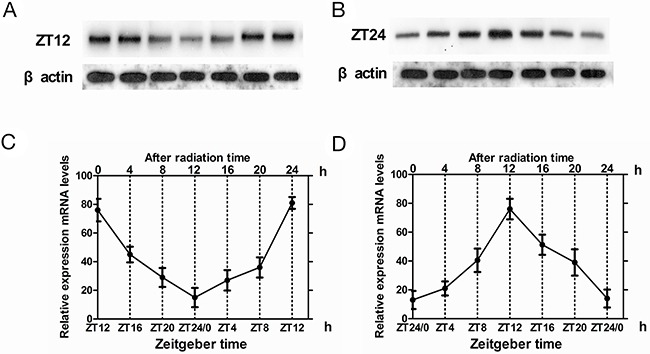
Cry2 mRNA and protein expression in normal brain tissues after irradiation **(A, B)** Western blot analysis of CRY2 protein in irradiated and non-irradiated normal brain tissues at ZT12 and ZT24 time points. **(C, D)** QRT-PCR analysis of cry2 mRNA expression in irradiated and non-irradiated normal brain tissues at ZT12 and ZT24 time points. Data are the mean and standard deviation of three independent experiments.

Next, we determined the effects of irradiation (15Gy) on Cry2 expression in glioma and normal brain tissues. In normal brain tissues, *cry2* mRNA levels were high at ZT12 and low at ZT24 time points. We observed no significant changes due to irradiation at either ZT12 (*t* = –1.386, p>0.05) or ZT24 (*t* = –1.386, p>0.05; Figure 2A, 2B). Similarly, cry2 mRNA expression was rhythmic in normal brain tissue in spite of the irradiation (F=8.67, p<0.001; Figure 2C, 2D). Similar results were observed for CRY2 protein expression in normal tissues at ZT12 (*t* = –5.276, *p* < 0.001) and ZT24 (*t* = –6.599, *p* < 0.001) (Figure 2C, 2D). CRY2 protein expression was rhythmic upon irradiation in normal brain tissues (F=10.81, p<0.001; Figure 2C, 2D).

**Figure 3:**
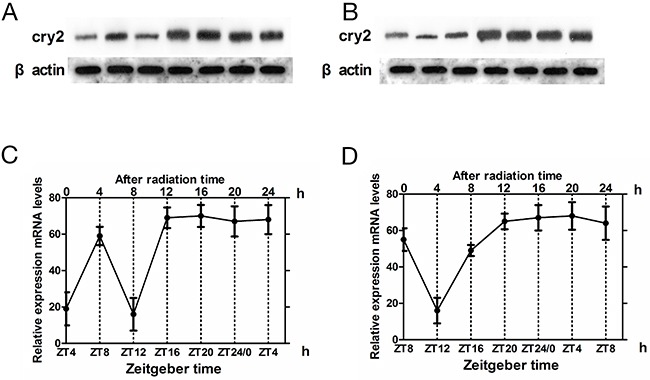
Cry2 mRNA and protein expression in glioma tissues after irradiation **(A, B)** Western blot analysis of CRY2 protein in irradiated and non-irradiated glioma brain tissues at ZT4 and ZT8 time points. **(C, D)** QRT-PCR analysis of cry2 mRNA expression in irradiated and non-irradiated glioma brain tissues at ZT4 and ZT8 time points. Data are the mean and standard deviation of three independent experiments.

In glioma tissues, irrradiation induced high cry2 mRNA expression and disappearance of rhythmicity (F=0.34, P>0.5). The levels of *cry 2* mRNA were higher in the irradiated glioma group than in the control glioma group at ZT8 (*t* = –5.135, p< 0.001) and ZT4(*t* = –4.464, p < 0.001), which represent high and low cry2 expression time points for glioma brain tissues, respectively (Figure 3).

Upon irradiation, CRY2 protein expression increased in glioma tissues at ZT4 (*t* = –5.276, p < 0.001) and ZT8 (*t* = –6.599, p < 0.001), with loss of rhythmicity (F=0.57, p >0.5; Figure 3). These results were similar to cry mRNA patterns in the glioma tissues.

### *CRY 2* levels correlate with glioma cell proliferation

**Figure 4:**
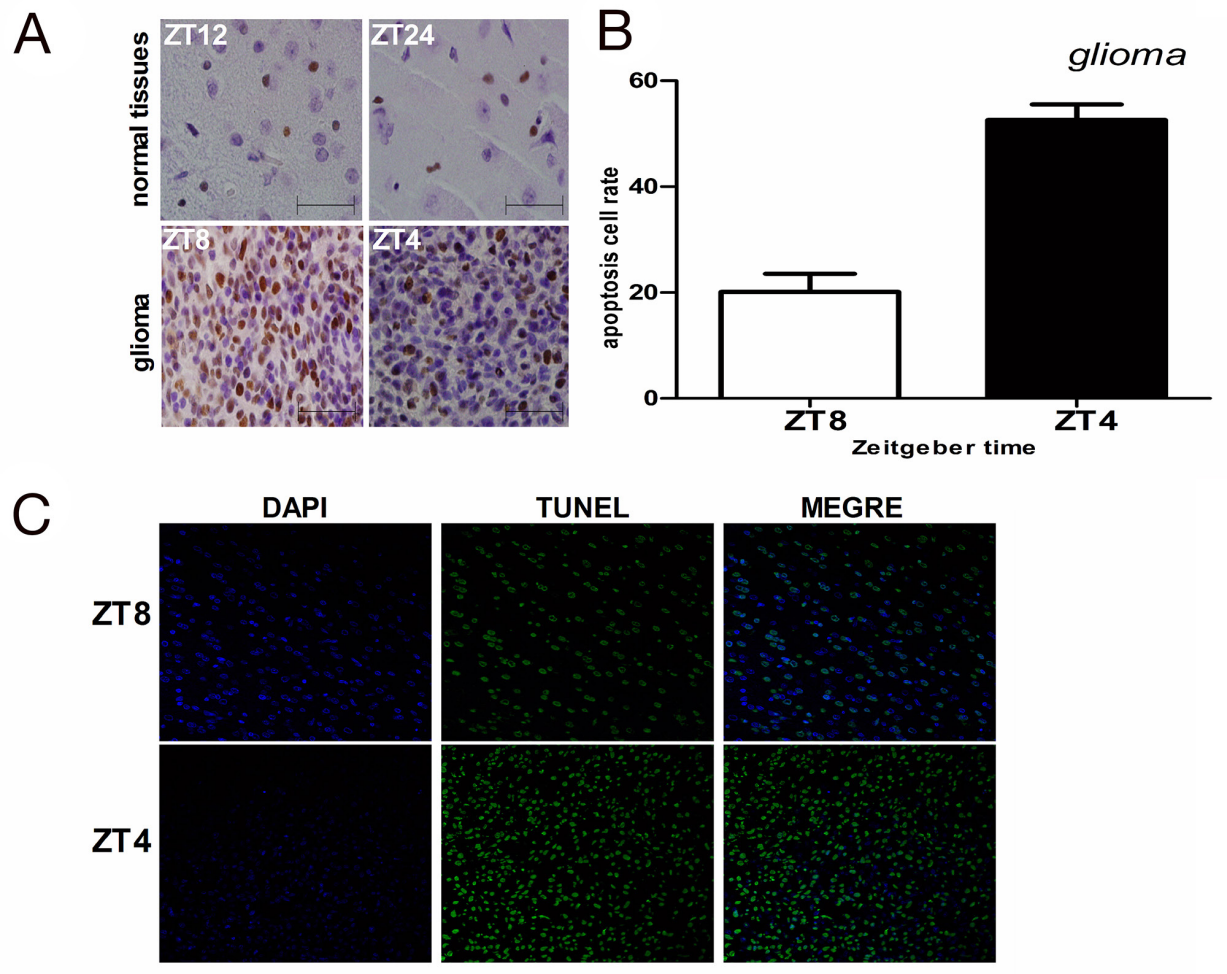
Cell proliferation and apoptosis in glioma and normal brain tissues after irradiation **(A)** Immunohistochemical analysis of PCNA-positive cells in irradiated glioma and normal brain tissues at high (ZT8 in glioma and ZT12 in normal) and low (ZT4 in glioma and ZT24 in normal) *cry*2 expression time points. **(B, C)** Immunohistochemical analysis of TUNEL-positive cells (apoptotic) in irradiated glioma and normal brain tissues at high (ZT8 in glioma and ZT12 in normal) and low (ZT4 in glioma and ZT24 in normal) *cry*2 expression time points.

Next, we measured the effects of irradiation (15Gy) on cell proliferation in glioma and normal brain tissues when *CRY2* levels were either high or low by PCNA immunohistochemical analysis. In glioma brain tissues, the proportion of proliferating cells (PCNA^+ve^) were 69.4% at ZT8 when *cry2* expression was maximal compared to 34.7% at ZT4 when cry2 expression was lowest (p<0.01; Figure 4A). In normal brain tissues, the proportion of proliferating cells were similar at ZT12 (12.8%) and ZT24 when *cry2* levels were high and low, respectively (Figure 4A; p>0.05). Therefore, aberrantly increased proliferation in glioma tissues correlated with *Cry2* mRNA and protein levels.

### High *CRY2* levels negatively correlate with glioma cell apoptosis

We performed TUNEL assay to analyze apoptosis in glioma and normal brain tissues after irradiation when CRY2 levels were either high or low. Apoptosis in glioma tissue correlated with the level of *cry2* expression. In glioma tissues, the proportion of apoptotic cells was lower (20.1%) at ZT8 when *cry2* expression was high compared to 52.6% at ZT4 when cry2 expression was lower (Figure 4B, 4C; p<0.001). In contrast, the proportion of apoptotic cells at ZT12 and ZT24 that denoted high and low cry2 expression time points were similar at 17.8% and 15.9%, (Figure 4B, 4C; p>0.05). These data suggested that cry2 expression correlated with sensitivity to apoptosis in glioma cells after irradiation

### Effects of irradiation on p53 and p73 expression in glioma tissues

**Figure 5:**
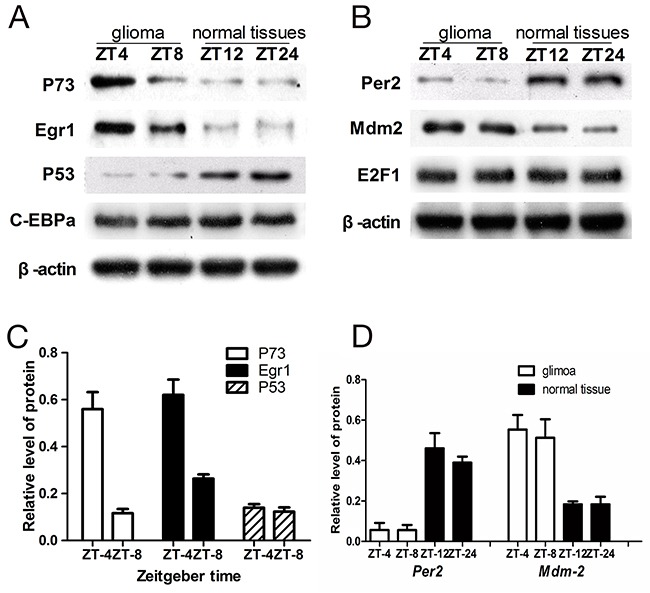
Western blot analysis of p73, Egr1, p53, Per2, MDM2, C-EBPα and E2F1 in glioma and normal tissues **(A)** Relative expression of p73, Egr1, p53, C-EBPα in comparison to β-actin in glioma and normal brain tissues. **(B)** Relative expression of per2, mdm-2 nd E2F1 in comparison to β-actin in glioma and normal brain issues. The values are from 3 replicate experiments and error bars denote SEM. **(C)** The ratios of the p73, Egr1, p53 signals to the β-actin signal in the glioma were arbitrarily set at 1.0. **(D)** The ratios of the per2, mdm-2 signals to the β-actin signal in the glioma and normal brain tissues. The error bars indicate the standard errors of the means (SEMs; n=3).

Next, we analyzed the expression levels of p73, Egr1, p53, Per2, Mdm2, C-EBPα and E2F1by western blot (Figure 5). In glioma tissues, p53 expression was similar at ZT8 and ZT4 (t=-0.501, p>0.5), whereas p73 levels were higher at ZT4 compared to ZT8 (t=-2.347, p<0.01) time points. Similarly, Egr1 levels were higher at ZT4 compared to ZT8 (t=-2.016, p<0.01; Figure 5). In normal brain tissues, p53, p73 and Egr1 were not significantly altered by irradiation treatment at ZT12 or at ZT24 (Figure 5). Also, C-EBPα and E2F1 levels were not altered by irradiation in both glioma and normal brain tissues.

In glioma tissues, the Per2 levels were similar at ZT4 and ZT8 (t=-0.144, p>0.5). Moreover, Per2 levels in glioma tissues at ZT8 were lower compared to normal brain tissues at ZT12 (t=-3.218, p<0.01) when cry2 expression was highest in both samples. Similarly, lower Per2 levels were observed in glioma tissues at ZT4 compared to ZT24 in normal brain tissues when cry2 expression was lowest in both (t=-2.508, p<0.01; Figure 5).

The expression of MDM2 at ZT4 and ZT8 in glioma tissues were comparable (t=-0.098, p>0.5). However, MDM2 levels in glioma tissues at ZT8 were higher than at ZT12 in normal brain tissues when cry2 expression was highest (t=-5.047, p<0.001; Figure 5). Similarly, MDM2 levels were higher at ZT4 in glioma tissues compared to ZT24 in normal brain tissues when cry2 expression was lowest (t=-4.361, p<0.001; Figure 5).

## DISCUSSION

In this study, we demonstrate aberrant expression and circadian rhythm of the clock gene *cry2* in the gliomas compared to normal brain tissue. The *cry2* expression shows a periodicity of 8h in glioma tissues compared to 24h in normal brain tissues. The aberrant periodic oscillation in *cry2* expression in glioma tissues indicates altered circadian rhythm, which is regulated by SCN. Cultured astrocytes show circadian oscillation of clock genes [19] with sustained rhythms when co-cultured with explants of adult SCN, but not with cortical explants [20]. Interestingly, 25% of the cells within the SCN are astrocytes. Moreover, aberrant clock gene expression may be associated with the occurrence of glioma [21–22].

In our study, we irradiated normal tissues at ZT12 and ZT24 and glioma tissues at ZT4 and ZT8 when CRY2 expression was lowest and highest, respectively. We observed that *CRY2* levels in irradiated glioma tissue were significantly higher compared to untreated glioma, whereas irradiation did not change CRY2 levels in normal brain tissues. Previous studies showed that irradiation upregulated transcription of a number of murine clock genes [11], but not *CRY2.* Our study shows that x-ray irradiation increases *CRY2* expression in glioma tissues.

To determine the relevance of these changes in *cry2* expression on glioma, we measured proliferation and apoptosis of glioma cells when rats were irradiated when *Cry2* mRNA and protein levels were high and low. We observed increased proliferation and diminished apoptosis at ZT8 compared to ZT4. This suggested that high cry2 expression correlated with increased proliferation and decreased apoptosis.

*Cry* is the main repressor in the transcription-translation feedback loop that generates circadian rhythmicity in mice. Despite the epidemiological findings suggesting that disruption of the circadian clock predisposes humans to cancer [15, 23–24], the current data from mouse model systems is conflicting [11]. *Per2* mutations in mice disrupt the clock and predispose mice to cancers, whereas disruption of the clock by *Cry* mutations (which also affects the negative transcription-translation feedback loop of circadian clock like Per2) does not increase cancer risk [25]. Also, *clock* mutations that affect the positive transcription-translation feedback loop of circadian rhythm do not increase cancer risk [11]. These data suggest that general clock disruption per se does not predispose mice to cancers and that the core clock genes have unique functions. Nuri *et al.* demonstrated that downregulation of Cry2 in combination with p53-null mutations delayed progression of cancer [10]. Therefore, we postulate that deletion of Cry2 sensitizes transformed cells to p53 independent apoptotic signals.

Many DNA damaging agents initiate apoptosis. Ionizing radiations cause double-strand breaks (DSBs), which activates replication checkpoints to delay S phase progression and G2/M transition and irreparable damage activates apoptosis [26]. In mammalian cells with damaged DNA, the *p53* tumor suppressor and the *Rb* family of transcriptional repressors coordinate to downregulate proteins required for the G2/M transition, thereby arresting cells in G2 phase [26]. The p53 apoptotic pathway is one of the many pathways involved in apoptosis.

We observed that the high cry2 levels were associated with low apoptosis and without any alteration in p73 levels. In contrast, downregulation of cry2 increased apoptosis and increased p73 expression. These results suggest that *cry2* may play an important role in promoting carcinogenesis by suppressing apoptosis. Genotoxic agents induce apoptosis in tumor cells via p53 in most cases [10]. However, in p53-mutant cells, they rely on p73 to induce apoptosis [27]. In contrast to p53, p73 is upregulated by genotoxic agents mostly by transcriptional induction [27–28]. The p73 promoter contains binding sites for Egr1, C-EBPα and E2F1, of which Egr1 is controlled by the circadian clock [29]. E2F1 is regulated by the cell cycle with DNA damage–induced phosphorylation and acetylation stabilizing and enhancing its activity [30–32]. In most cell types, p73 expression is low in the absence of DNA damage [33]. We observed upregulation of p73 in glioma tissues with the low cry2 expression. This is compatible with a hypothesis that CRY2 controls Egr1 gene expression and maybe key to glioma growth and development.

There was an increase in p53 expression that was not expected after irradiation in glioma. The regulation of p53 is complicated and linked to many factors. One of the genes involved in posttranscriptional regulation of *p53* is *Mdm-2*, which mediates ubiquitination of p53 and further degradation by the proteasome pathway [34]. Per2 also modulates the stability of p53 and transcriptional activity in response to genotoxic stress [35]. Per2 transcriptionally modulates TP53 and directly associates with the C-terminus region of p53, thereby preventing Mdm2-mediated ubiquitination of p53 and subsequent proteasomal degradation [34]. Also nuclear shuttling of p53 was promoted by ectopic expression of Per2 and reduced by Per2 down-regulation [35]. Therefore, Per2 is a crucial factor that plays a key role in expression, degradation and nuclear shuttling of p53. Altouhgh the levels of MDM-2 were high and Per2 levels were low in the present study, the mechanism responsible for the observed decrease in p53 induction after radiation is not fully understood.

Our findings support previous studies that showed high expression of *Per2* after irradiation upregulated *p53*, thereby promoting apoptosis. Therefore, *Per2* act as tumor suppressors in gliomas and their high expression can induce cell cycle arrest and increase tumor sensitivity to x-rays through a p53-dependent mechanism. However, our study also suggests other mechanisms of inducting apoptosis in glioma by circadian genes.

In conclusion, our study demonstrates that Cry2 plays a significant role in cell cycle, apoptosis and proliferation of glioma cells.

## MATERIALS AND METHODS

### Cell culture

C6 glioma cells were grown in Dulbecco's Modified Eagle's Medium (DMEM, Hyclone, catalog no. SH30022.01B) medium supplemented with 10% fetal bovine serum (FBS, Hyclone, catalog no. SV30087.01) at 37°C and 5% CO_2_.

### Animal experiments

The animal studies were conducted in accordance with the guidelines instituted by the Animal Studies Committee Ethics of Biomedicine Research, First People's Hospital of Jingmen. Sprague-Dawley male rats (n=360, 120-150g) were obtained from Ningxia Medical University Experimental Animal Center. The rats were housed in a standard light/dark cycle of 12h: 12h at 24±1 °C. In this study, times are reported as Zeitgeber time (ZT), or hours after light onset. Thus, ZT0 denotes time when lights were turned on and ZT12 denotes time when lights were turned off. The animals adapted to the 12:12h light/dark cycle for 4 weeks before the experiments. The rat glioma model was established according to protocol by Watanabe *et al* [16] and adapted to the 12:12h light/dark cycle for 2 weeks. Animals were anesthetized for irradiation and perfusion procedures with an intraperitoneal injection of 50mg/kg sodium pentobarbital. After 48 h, the irradiated animals were sacrificed at each every 4h and tissue was collected.

We first examined the mRNA expression of *Cry2* genes in rat glioma and normal tissues over 24h by sacrificing 10 rats each every 4h to obtain ZT4, ZT8, ZT12, ZT16, ZT24 (ZT0) time points. Tumor and normal brain tissues were rapidly removed and frozen in liquid nitrogen and processed further to obtain mRNA and protein for analysis by qRT-PCR and western blotting, respectively. Another 60 rats were sacrificed by anesthesia (100mg/kg) and perfused with 4% paraformaldehyde. The brains were fixed overnight in 10% phosphate-buffered formalin (PBF), rinsed in 70% ethanol, and paraffin-embedded.

To determine the expression of *Cry2* in glioma and normal tissues after irradiation, tumor-bearing rats (n = 200) were randomized into four groups and subjected to 15Gy irradiation at times when *cry 2* mRNA levels were high (at ZT12 for normal tissues and ZT8 for glioma) and low (at ZT24 for normal tissues and ZT4 for glioma). After irradiation, we examined the mRNA expression of *Cry2* genes in rat glioma and normal tissues over 24 h by sacrificing 10 rats every 4 h to obtain the ZT4, ZT8, ZT12, ZT16, and ZT24 (ZT0) time points. The tumor and contralateral normal brain tissues were rapidly removed and frozen in liquid nitrogen and used to determine *Cry2* mRNA and protein levels by qRT-PCR and western blotting, respectively.

To determine the effects of irradiation on glioma and normal brain tissues at high and low *Cry2* mRNA time points, tumor-bearing rats (n = 40) were randomized into 4 groups, and 20 rats each were administered a single 15Gy dose of irradiation (Varian 2100C/D, USA) at ZT4, ZT8, ZT12, ZT24 (ZT0). Then, 8h after irradiation, rats were killed by anesthesia (100 mg/kg) and perfused with 4% paraformaldehyde [17–18]. The brains were fixed overnight in 10% phosphate-buffered formalin (PBF), rinsed in 70% ethanol, and paraffin-embedded.

### Quantitative real time-PCR

Total RNA from glioma and normal brain tissues was extracted using Trizol reagent (Invitrogen, catalog no. 15596026). RNA (1μg) was reverse-transcribed using the cDNA synthesis kit (Invitrogen, catalog no. 11917020) following the manufacturer's instructions. For PCR, the following Cry2 and β-actin primers were used: *cry2* sense, 5-AGTCAAGCAAACCTGGAAGG-3; *cry2* antisense, 5-AATCATCCTGCTACCCGAAG-3; *β-actin*sense, 5-CC CATCTATGAGGGTTACGC-3; *β-actin* antisense, 5-TTT AATGTCACGCACGATTTC-3. For each set, six replicate samples were run with one replicate used for calibration. Expression of *cry2* mRNA was determined by the 2^-ΔΔCt^ analysis relative to β-actin.

### Immunohistochemical analysis of Cry2

Paraffin-embedded tissue sections (4μm) were deparaffinized on poly-1-lysine coated slides. The sections were pressure cooked with EDTA for 2.5 min, cooled and then incubated with 3% H_2_O_2_ for 10 min to block endogenous peroxidase followed by incubation with gradient alcohol concentrations to hydrate the tissues. The sections were then washed thrice in phosphate buffered saline (PBS) for 2 minutes each. After being bathed in PBS, tissue sections were incubated with primary Cry2 antibody (1:400, Santa Cruz Biotechnology, CA, USA) for 2h at 37^°^C. The slides were then washed thrice with PBS and incubated with anti-rabbit secondary antibody (PV-6001, Zhongshan Goldenbridge Biotechnology Co., China) at 37^°^C for 30 minutes, then thoroughly washed thrice with PBS. Cry2 expression was detected by developing the tissue samples with diaminobenzidine (DAB, Zhongshan Goldenbridge Biotechnology Co. China.) and counterstained with haematoxylin. Stained tissue sections were evaluated by a pathologist and an investigator who were both blind to diagnosis and the samples were scored as positive or negative.

Positive stained cells that had yellow staining in the nucleus were quantified in one high power field (400x). The scoring system for tumor cells was as follows: grade 0, no positive cells; grade 1, 1-25% positive cells; grade 2, 26 -50% positive cells; grade 3, 51-75% positive cells and grade 4, 75-100% positive cells. The staining intensity was also graded, with no coloration graded as 0, light yellow as 1, yellow as 2, and brown as 3. Finally, the two scores were multiplied and samples with a value greater than or equal to 2 were counted as positive.

### Immunohistochemical analysis of cell proliferation

Cellular proliferation in glioma and normal brain tissue samples was determined by immunohistochemical staining of proliferating cell nuclear antigen (PCNA). Paraffin-embedded tissue sections (4μm) were first blocked with goat serum to reduce non-specific binding and then incubated with monoclonal mouse anti PCNA antibody (1:100 dilution, Boster, catalog no. BM0104, Wuhan, China) overnight at 4^°^C. After three washes with PBS (pH7.2), a secondary antibody kit (Bioss, catalog no. SP-0024, Beijing, China) was used according to the manufacturer's instructions and developed with diaminobenzidine (DAB). Slides were counterstained with hematoxylin for 3 min, dehydrated through a graded series of ethanol concentrations and the samples were immersed in xylene for 5 min and the slides examined by light microscopy (Olympus BX-61). The primary antibody was replaced with PBS for negative control.

### TUNEL assay for apoptosis

Apoptotic cells in tumor and normal brain tissue sections were analyzed by terminal deoxynucleotidyl transferase-mediated deoxyuridine triphosphate-biotin nick end-labeling (TUNEL, Roche, 11684817910) assay according to the manufacturer's instructions. After deparaffinization, sections were treated with proteinase K (20 mg/ml in 10 mM Tris/HCl, pH 7.4-8.0) for 30 min at 37°C. Then the sections were incubated with TUNEL reaction mixture for 60 min at 37°C in a humidified atmosphere in the dark. The slides were rinsed thrice with PBS and mounted with Vecta Shield mounting medium containing DAPI (Vector Laboratories, catalog no. H-1200), The stained sections were analyzed by laser scanning confocal microscope (Olympus FV1000 Viewer) at 450-500nm excitation and 515-565nm (green) detection wavelengths.

### Western blotting

The tumor and normal brain tissue samples were lysed in cold RIPA lysis buffer with protease inhibitors and centrifuged. The protein supernatant was quantified by the bicinchoninic acid protein assay (Pierce, Rockford, Illinois). Equal amounts of proteins were then separated on SDS-PAGE and then transferred onto PVDF membranes. After blocking with 5% skimmed milk, the blots were incubated with primary antibodies against CRY2, P53, P73, Per2, Mdm2, Egr1, C-EBPα, E2F1 and β-actin. The immunoblots were developed with Supersignal West Pico chemiluminescence substrate (Pierce, Rockford, Illinois, USA) and and analyzed with the Bio-Rad imaging system (Bio-Rad, Hercules, California, USA).

### Statistical analysis

QRT-PCR and Western Blotting results are reported as mean ± s.d and the differences between samples were compared using the ANOVA test and Student's *t*-test. The circadian changes in *cry* 2 was analyzed using the methods for cosinor-rhythmometry, as described by Nelson et al. The model fit was then tested using the GLM procedure in the SAS statistical software package. The Pearson chi-square test was used to compare apoptosis and proliferation results between different groups. *P<* 0.05 was considered statistically significant.

## Abbreviations and acronyms

Per1: Period1
Per2: Period2
SCN: Suprachiasmatic nuclei
ZT: Zeitgeber time
Cry2: Cryptochrome2
*BMAL1*: Brain-muscle Arnt-like protein 1
*Npas2*: Neuronal PAS 2
DSBs: Double-strand breaks
IR: Ionizing Radiation
FBS: Fetal bovine serum
Mdm2: murine double minute 2
DMEM: Dulbecco's Modified Eagle's Medium
PBF: Phosphate-buffered formalin
PCNA: Proliferating cell nuclear antigen
PBS: Phosphate-buffered saline
